# Genome-wide survey of the bipartite structure and pathogenesis-related genes of *Neostagonosporella sichuanensis*, a causal agent of Fishscale bamboo rhombic-spot disease

**DOI:** 10.3389/fmicb.2024.1456993

**Published:** 2024-09-18

**Authors:** Lijuan Liu, Chunlin Yang, Fang Liang, Chengsong Li, Qian Zeng, Shan Han, Shujiang Li, Yinggao Liu

**Affiliations:** ^1^College of Forestry, Sichuan Agricultural University, Chengdu, China; ^2^National Forestry and Grassland Administration, Key Laboratory of Forest Resources Conservation and Ecological Safety on the Upper Reaches of the Yangtze River and Forestry Ecological Engineering in the Upper Reaches of the Yangtze River Key Laboratory of Sichuan Province, College of Forestry, Sichuan Agricultural University, Chengdu, China

**Keywords:** *Neostagonosporella sichuanensis*, genomic resources, *Phaeosphaeriaceae*, repeat-induced point mutation, genome evolution, pathogenicity-related genes

## Abstract

Bamboo resources have garnered significant global attention due to their excellent capacity for regeneration and high yield. Rhombic-spot disease, a substantial threat to fishscale bamboo (*Phyllostachys heteroclada*), is primarily caused by *Neostagonosporella sichuanensis*. This study first reported the genome assemblies and characteristics of two *N. sichuanensis* isolates using PacBio and Illumina sequencing platforms. The genomes of *N. sichuanensis* strain SICAUCC 16–0001 and strain SICAUCC 23–0140, with sizes of 48.0 Mb and 48.4 Mb, respectively, revealed 10,289 and 10,313 protein-coding genes. Additionally, they contained 34.99 and 34.46% repetitive sequences within AT-rich regions, with notable repeat-induced point mutation activity. Comparative genome analysis identified 1,049 contracted and 45 expanded gene families in the genome of *N. sichuanensis*, including several related to pathogenicity. Several gene families involved in mycotoxin metabolism, secondary metabolism, sterol biosynthesis and transport, and cell wall degradation were contracted. Compared to most analyzed necrotrophic, hemibiotrophic, and phaeosphaeriacous pathogens, the genomes of two *N. sichuanensis* isolates exhibited fewer secondary metabolite enzymes, carbohydrate-active enzymes, plant cell wall degrading enzymes, secreted proteins, and effectors. Comparative genomics analysis suggested that *N. sichuanensis* shares more similar characteristics with hemibiotrophic pathogens. Based on single carbon source tests, *N. sichuanensis* strains demonstrated a higher potential for xylan decomposition than pectin and cellulose. The proportion of cell wall-degrading enzyme effectors occupied a high proportion of the total effectors of the *N. sichuanensis* genomes. These findings provide valuable insights into uncovering the pathogenesis of *N. sichuanensis* toward the efficient management of rhombic-spot disease of fishscale bamboo.

## 1 Introduction

*Neostagonosporella sichuanensis*, belonging to the genus *Neostagonosporella* with only two recorded species (*N. sichuanensis* and *N. bambusicola*) (Yang et al., 2019; Dissanayake et al., 2022), is the primary pathogen of rhombic-spot disease in fishscale bamboo (*Phyllostachys heteroclada*), an economically important culm and shoot-producing bamboo species widely cultivated in many provinces of China (Zhou et al., 2017). The life cycle and epidemiology of *N. sichuanensis* have been well understood (Liu et al., 2022). *N. sichuanensis* can infect the culms, branches, twigs and exposed rhizomes, and result in devastating diseases to fishscale bamboo. Typically, dense oval, diamond-shaped, nearly diamond-shaped, and irregular lesions are covered in infected tissues, resulting in the inadequate growth, and eventual death in some cases, of bamboo plants. Our previous study (Liang et al., 2024) identified an effector, NsCFEM1, a vital virulence factor affecting *N. sichuanensis* growth and pathogenicity. However, since relatively few studies have been published on *N. sichuanensis*, the molecular mechanisms behind its pathogenicity remain unclear. Highly continuous and complete genome assemblies and accurate genome annotations will be essential for identifying structural variations, gene mapping, and cloning, as well as for understanding mechanisms of pathogenicity.

*Neostagonosporella sichuanensis* is located in the family of *Phaeosphaeriaceae*, one of the most speciose families in the order *Pleosporales*, consists of 84 genera and more than 1,000 species that exist in various ecosystems, including terrestrial, freshwater, marine, and mangrove habitats (Tennakoon et al., 2020; Wijayawardene et al., 2022). Members of this family exhibit diverse lifestyles. They are widely distributed and are typically saprobic, endophytic, and symbiotic, but occasionally pathogenic to various plants and humans (Zhang et al., 2019). Some of them are among the most dominant pathogenic organisms in plant diseases causing significant losses in quality and yield. For instance, *Parastagonospora nodorum*, a highly concerning necrotrophic pathogen in the *Phaeosphaeriaceae* family, has been frequently associated with significant economic losses in crops due to wheat smut (John et al., 2022). *Setophoma terrestris*, a saprobe inhabiting ubiquitously found in soil, sometimes turns into a causal agent of pink root rot in various crops (Yoshida, 2021). Additionally, numerous species have been identified as pathogens causing leaf spots on various plant hosts, such as *Parastagonosporella* (Bakhshi et al., 2019), *Phaeosphaeriopsis* (Thambugala et al., 2014), and *Setophoma* (Liu et al., 2019). Next-generation sequencing technologies continue to advance, thereby providing increasingly well-characterized and cost-effective fungal genomes, which has increased the pace of plant pathogen research and substantially advanced our understanding of different pathogens. However, as of May 2024, according to the National Center for Biotechnology Information (NCBI) database (Sayers et al., 2022) and in the Joint Genome Institute’s (JGI) MycoCosm (Nordberg et al., 2014), only nine whole-genome resources of phaeosphaeriacous species were sequenced and available, of which five are reported as pathogens, including *Ophiobolus disseminans*, *P. nodorum*, *Stagonospora* sp., *Setomelanomma holmii*, and *S. terrestris*. Numerous genomes of phaeosphaeriacous pathogens remain unexplored.

Pathogenic fungi employ diverse strategies to infect their host plants, including biotrophic, hemibiotrophic and necrotrophic strategies (Lo Presti et al., 2015). Secondary metabolic toxins and cell wall degradation enzymes are usually necessary for hemibiotrophic and necrotrophic pathogens in their necrotrophic stage to kill plant cells for nutrients, in contrast to biotrophic pathogens (Lo Presti et al., 2015). The survey results showed that the secondary metabolites biosynthetic enzymes and carbohydrate-active enzymes (CAZymes) of necrotrophic and hemibiotrophic pathogens are generally significantly enlarged compared to those of biotrophic pathogens (Wang et al., 2018; Zhao et al., 2013). Therefore, the number of secondary metabolite biosynthetic enzymes and CAZymes can be used to define the different lifestyles of pathogens. The available genome resources bring considerable convenience to unprecedented insights into genome composition. Among the five phaeosphaeriacous pathogens mentioned above, only *Parastagonospora nodorum* was reported as a necrotrophic pathogen (John et al., 2022), but lifestyles of the remaining four genomes-available pathogens are still unknown, including *Ophiobolus disseminans*, *Stagonospora* sp., *Setomelanomma holmii*, and *Setophoma terrestris*. The same is true of *N. sichuanensis*. With the help of genomic resources, we can predict their potential lifestyles, which will lay the foundation for a more systematic understanding of the pathogenic mechanism of these pathogens.

Herein, in this study, we report two high-quality reference genome assemblies of *Neostagonosporella sichuanensis* SICAUCC 16–0001 and *N. sichuanensis* SICAUCC 23–0140 based on sequence data from whole-genome shotgun (WGS) sequencing platforms of PacBio Sequel IIe and Illumina NovaSeq PE150 technologies. The objectives of this study were to: (1) characterize and annotate the two genomes, and systematically compare the genomic structural features between *N. sichuanensis* and other phaeosphaeriacous species; (2) confirm the position of *N. sichuanensis* and other phaeosphaeriacous species in *Phaeosphaeriaceae* (*Pleosporales*) using phylogenomic analysis, calculate the divergence time of *N. sichuanensis* and other phaeosphaeriacous species, and characterize the expansion and contraction gene families of the genomes; and (3) understand variations in pathogenesis-related gene contents between *N. sichuanensis* and other phaeosphaeriacous pathogens, as well as fungi with different lifestyles, and predict the possible lifestyles of *N. sichuanensis* and other four phaeosphaeriacous pathogens (*Ophiobolus disseminans*, *Stagonospora* sp., *Setomelanomma holmii*, and *Setophoma terrestris*) with unknown lifestyles.

## 2 Materials and methods

### 2.1 Sample collection, DNA extraction, genome sequencing, and assembly

*Neostagonosporella sichuanensis* strain SICAUCC 16–0001 and SICAUCC 23–0140 were initially isolated from diseased branches and stems of fishscale bamboo, respectively, by the National Forestry and Grassland Administration Key Laboratory of Forest Resources Conservation and Ecological Safety on the Upper Reaches of the Yangtze River, College of Forestry, Sichuan Agricultural University, Chengdu, China. They were collected from Zhougongshan Town (29°50′8.56″N 103°2′59.87″E, collection date: 4 August 2016), Yanchang Town (103°4′44.4″E, 29°43′33.95″N, collection date: 1 March 2022) in Ya’an City, Sichuan Province, China, respectively. The two strains had similar morphological characteristics on potato dextrose agar (PDA) and there was no significant difference in their virulence (Supplementary Figure S1). The two strains were cultured for 10 days on PDA medium at 25°C before being cultured in potato dextrose broth (PDB) at 25°C with agitation at 180 rpm for 14 days. Next, fresh mycelia samples of both strains were harvested and sent to Shanghai Majorbio Bioinformatics Technology Co. Ltd. and Novogene Bioinformatics Technology Co. Ltd. in Beijing for genome sequencing, respectively. Genomic DNA was extracted using the sodium dodecyl sulfate method (Lim et al., 2016), assessed by agarose gel electrophoresis, and quantified on a Qubit® 2.0 Fluorometer (Thermo Fisher Scientific, Waltham, MA, United States). The concentration and total amount of DNA samples were 81.8 ng/μL and 16.36 μg for strain SICAUCC 16–0001, and 163 ng/μL and 45.31 μg for strain SICAUCC 23–0140, respectively. We found the ratios between the absorbance at 260 nm and 280 nm (A260/280) and A260/230 of the DNA samples to be 1.80 and 2.07 for strain SICAUCC 16–0001, and 1.80 and 2.41 for strain SICAUCC 23–0140, respectively.

### 2.2 Genome sequencing, assembly, and assessment

Single Molecule Real-Time sequencing libraries were generated with an insert size of 20 kb using the SMRTbellTM Template Prep Kit v2.0 (Pacbio, United States) following the manufacturer’s recommendations. Nanopore sequencing DNA samples were fragmented to 350 bp, and the DNA libraries were constructed using the NEBNext® Ultra™ DNA Library Prep Kit for Illumina (NEB, USA). Later, a Qubit 2.0 Fluorometer (Thermo Fisher Scientific, Waltham, MA, USA) and an Agilent 2,100 Bioanalyzer (Agilent Technologies, Santa Clara, California, USA) were used to analyze the concentration and size of the library, respectively. Then, the whole genomes of the two strains were sequenced using a WGS strategy on the PacBio Sequel IIe and the Illumina NovaSeq PE150 sequencing platforms. Low-quality reads or adapters from Illumina and PacBio sequencing were filtered using FastP v0.23.0 with the default parameters (Chen, 2023). Pacbio clean reads were then assembled using Falcon v0.3.0 (Wick et al., 2017; Reiner et al., 2018), corrected three times using Racon v1.4.13 based on itself (Vaser et al., 2017), and corrected three times using Pilon v1.22 (Walker et al., 2014) based on Illumina clean reads. The completeness of the final assembly genomes was assessed using BUSCO v.5.4.7 (Simão et al., 2015).

### 2.3 RNA sequencing and analysis

Mycelia samples used for total RNA extraction of *Neostagonosporella sichuanensis* SICAUCC 16–0001 were hyphae collected in PDA medium and cultured at 25°C for 7 days. These collected samples were immediately frozen in liquid nitrogen and stored in the laboratory at −80°C until RNA extraction. Total RNAs were extracted using Trizol reagent (Invitrogen, Carlsbad, CA, United States, 15596026) following the single-step method (Chomczynski and Sacchi, 1987) before DNA digestion with DNaseI. RNA quality, integrity, and concentration were determined on the Nanodrop™ One^C^ Spectrophotometer [Thermo Fisher Scientific, Waltham, MA, United States), by agarose gel electrophoresis (1.5% (w/v)], and on a Qubit 3.0 Fluorometer using Qubit™ RNA Broad Range Assay Kit (Life Technologies, Q10210), respectively. The A_260/280_ ratios of the three RNA replicates were 2.05, 2.14, and 2.11, respectively. RNA concentrations of them were 99 ng/μL, 120 ng/μL, and 85 ng/μL, with total RNA contents of 8.98 μg, 11.55 μg, and 6.07 μg. All RNA quality number and RNA integrity number ratios (RQN/RIN) were 8.9 and examined by LabChip GX Touch. High-throughput cDNA libraries were prepared using the KC-DigitalTM Stranded mRNA Library Prep Kit for Illumina® (Catalog No. DR08502, Wuhan Seqhealth Co., Ltd., China) and sequenced on a Novaseq 6,000 PE150 sequencer (Illumina). High-quality sequences were obtained by FastQC v0.23.0.^1^ RNA-Seq data were mapped to *N. sichuanensis* using STAR v2.5.3a (Dobin et al., 2013) with default parameters, and SAMTools v1.13 (Danecek et al., 2021) was used to evaluate and merge read alignments. The genome-guided transcriptome of *N. sichuanensis* was then assembled using trinityrnaseq-v2.15.1 (Grabherr et al., 2011) with the “--jaccard_clip and --genome_guided_max_intron 1,000” options due to the high gene density and short intron length distribution of fungal species.

### 2.4 Gene prediction

The *de novo* repeats library was constructed using RepeatModeler v2.0.4 (Flynn et al., 2020) and then identified using RepeatMasker v4.1.5 (Tempel, 2012) for the repeat sequences. For *Neostagonosporella sichuanensis* SICAUCC 16–0001, MAKER3 v3.01.04 (Cantarel et al., 2008) was used to predict the gene structures based on assembled transcripts. The obtained gene models were then used to train AUGUSTUS v3.5.0 (Mario et al., 2008). GeneMark-ES v4.62 (Ter-Hovhannisyan et al., 2008) was also used to predict the gene structures. The predicted gene models from AUGUSTUS and GeneMark-ES, as well as the homology proteins were combined in MAKER3 v3.01.04. Since there are only nine available genome resources in the family of phaeosphaeriacous, all of them were analyzed as homology proteins for gene prediction, including *Ampelomyces quisqualis* HMLAC05119, *Stagonospora* sp. SRC1lsM3a, *Setomelanomma holmii* CBS 110217, *Ophiobolus disseminans* CBS 113818, *Parastagonospora nodorum* SN15, *Leptosphaeria microscopica* UNIPAMPA013, *Paraphoma chrysanthemicola* MPI-SDFR-AT-0120, *Phaeosphaeria poagena* MPI-PUGE-AT-0046c, *Setophoma terrestris* DSE6109. These genomes were downloaded from the NCBI genome database and the DOE JGI Genome Portal and then their redundancy sequences were removed by cd-hit v4.8.1 (Fu et al., 2012) before being used for gene prediction. SICAUCC 23–0140 genes were performed *de novo* prediction using MAKER3 v3.01.04 combining the AUGUSTUS and GeneMark-ES gene models and homology-based prediction using GeneWise v2.4.1 (Birney et al., 2004) based on homologous protein sequences of strain SICAUCC 16–0001. The two outcomes were then incorporated using EVM (r2012-06-25) and confirmed with PASA v2.5.3 (Haas et al., 2011) in the second round to obtain the consensus coding genes. Finally, tRNAs were predicted using tRNAscan-SE v2.0.12 (Chan et al., 2021).

### 2.5 Genome annotation and analysis

The predicted protein sequences were functionally annotated using several databases with a threshold for the *E*-value set to ≤1 × 10^−5^, including NR (Non-Redundant Protein Database) (Sayers et al., 2022), Pfam (Mistry et al., 2021), KOG (EuKariotic Orthologous Group database) (Galperin et al., 2014), GO (Gene Ontology databases) (Aleksander et al., 2023), KEGG (Kyoto Encyclopedia of Genes and Genomes database) (Moriya et al., 2007), and the eggNOG (evolutionary genealogy of genes: Non-supervised Orthologous Groups) database (Huerta-Cepas et al., 2019). Pathogen lifestyles were predicted using CATAStrophy v0.1.0 (Hane et al., 2020) with the dbCAN v10 database (Zhang et al., 2018). OcculterCut v.1.1 (Rouxel et al., 2011) program was then used to calculate the GC content distributions of each genome. The RIPper program (van Wyk et al., 2021) was used to calculate RIP indices while D-Genies v1.5.0 (Cabanettes and Klopp, 2018) was used to display the dot-plot graphs of genome sequence alignments with MashMap v2.0.

Secondary metabolite gene clusters were determined using the antiSMASH fungal version website^2^ (Blin et al., 2023) with default parameters. The ketoacyl synthase (KS) domains of polyketide synthase (PKS) were detected and extracted using the NAPDOS web tool (Klau et al., 2022). A maximum likelihood (ML) phylogenetic tree of KS domains was constructed using MEGA v11 (Tamura et al., 2021). A phylogeny test was carried out using 1,000 bootstrap replications. The obtained tree was visualized and annotated using the Evolview webserver (Subramanian et al., 2019). CAZyme annotations were performed using the dbCAN2 database (Zhang et al., 2018) with the HMMER-based classification tool (Potter et al., 2018) with 1 × 10^5^ as the cutoff *E*-value. All heatmaps were created using TBtools v2.039 (Chen et al., 2023). The secretion signals of candidate secreted proteins were predicted using SignalP v5.0 (Almagro Armenteros et al., 2019) and those proteins with secretion signals were predicted in TMHMM v2.0 (Krogh et al., 2001), PredGPI (Pierleoni et al., 2008) and BUSCA web server (Savojardo et al., 2018), respectively. Only those proteins lacking a transmembrane domain or any potential GPI-anchor, and convincingly located in the extracellular space, were considered as potential secreted proteins. OrthoFinder v2.5.5 (Emms and Kelly, 2019) was used to cluster secreted and effector protein sequences into orthologous families. Representative protein sequences were then chosen from each family at random to determine phylogenetic distance. Principal coordinate plots were created for data visualization using the “pcoa” function in R and statistically significant differences between groups were determined using ANOSIM analysis (Clarke, 1993). Evenn was used to generate Venn diagrams and flower plots of the core families (Yang et al., 2024).

### 2.6 Phylogenomic analyses and genome sequence alignments

The proteomes used in the phylogenomic analyses were collected from the NCBI GenBank genome database and the Joint Genome Institute’s MycoCosm portal (Supplementary Table S1). The single-copy orthogroups (SCOs) within all species included in the analyses were detected with OrthoFinder v2.5.5 (Emms and Kelly, 2019). These SCOs were then aligned individually using MAFFT v7.520 (Katoh and Standley, 2013). The aligned sequences were trimmed and removed gaps using trimAl v1.4 (Capella-Gutiérrez et al., 2009). The alignments were then used as inputs to estimate a maximum likelihood tree with RAxML-NG v1.2.1 (Kozlov et al., 2019) using the LG + G8 + F model and 100 bootstrap replicates. The resulting tree was visualized using FigTree v1.4.0 (Rambaut, 2018). The divergence dating between species was determined using the PL method with r8s v1.71 (Sanderson, 2003). CAFE v5 (Mendes et al., 2020) was used to compute the expansion/contraction protein family. The orthologous secondary metabolite core genes and effectors were identified using the OrthoFinder algorithm in OrthoVenn3 (Sun et al., 2023) using an *E*-value of 1 × 10^−5^.

## 3 Results

### 3.1 Genome features of *Neostagonosporella sichuanensis*

We generated a clean sequencing data set of about 7.4 Gb for *Neostagonosporella sichuanensis* SICAUCC 16–0001 and 77.90 Gb for *N. sichuanensis* SICAUCC 23–0140, thus yielding almost 147-fold and 1,543-fold genome coverage, respectively. The genome sizes of the two strains were estimated to be ~50.50 Mb and ~ 50.56 Mb, respectively, based on the 17-mer depth distribution of sequenced reads (Supplementary Table S2). Draft genome sequences yielded a ~ 48.0 Mb (*N. sichuanensis* SICAUCC 16–0001) and ~ 48.4 Mb (*N. sichuanensis* SICAUCC 23–0140) genome assembly that covered ~95% and ~ 96% of the estimated genome size, and contained 428 scaffolds (N50, 0.2 Mb) and 28 scaffolds (N50, 2.2 Mb), respectively (Table 1). All clean Pacbio long reads of the two strains were mapped to the assembled genome sequences, exhibiting a higher alignment with a mapping rate of 97.7 and 98.2%, respectively. Further, 1,631 out of 1,709 (96.1%) and 1,659 out of 1,709 (97.0%) groups were found in the genomes of strain SICAUCC 16–0001 and SICAUCC 23–0140, respectively, according to BUSCO data from the *Ascomycota* lineage, suggesting a high coverage and a high degree of completeness (Supplementary Table S3).

**Table 1:** Statistical results of genome assembly and gene prediction for *Neostagonosporella sichuanensis* (SICAUCC 16–0001 and SICAUCC 23–0140).

Statistics	*N. sichuanensis* SICAUCC 16–0001	*N. sichuanensis* SICAUCC 23–0140
Depth of genome coverage (x)	147	1,543
Assembly genome (Mb)	48.0	48.4
Number of Scaffords	428	28
Scaffold N50_length (Mb)	0.2	2.2
Max Scaffold length (Mb)	0.7	3.1
GC content (%)	40.3	40.2
Predicted coding genes	10,289	10,313
Gene total length (bp)	14,668,272	14,593,407
Gene average length (bp)	1,425	1,415
Gene length/Genome (%)	30.55	30.39
GC Content in Gene Region (%)	52.96	52.97
Introns number per gene (%)	1.67	1.63
Exon number per gene (%)	2.69	2.65
tRNAs	128	137
Nr	9,715 (94.42%)	9,666 (93.73%)
Pfam	7,159 (69.58%)	7,097 (68.82%)
KOG	4,801 (46.66%)	4,736 (45.92%)
GO	5,796 (56.33%)	5,731 (55.57%)
KEGG	4,018 (39.05%)	3,996 (38.75%)
eggNOG	8,911 (86.61%)	8,829 (85.61%)

Approximate 20.36 Gb of clean RNA sequencing (RNA-Seq) data of *Neostagonosporella sichuanensis* SICAUCC 16–0001 (Supplementary Table S4) was obtained to aid gene prediction. These data revealed a good alignment (98.02%) to the assembled genome (Supplementary Table S5). In addition, 10,289 and 10,313 protein-coding genes were defined in the strain SICAUCC 16–0001 and SICAUCC 23–0140, respectively, which was lower than the number for surveyed phaeosphaeriacous species (11,147 – 16,142) (Supplementary Table S6). Moreover, 1,637 out of 1,709 (96.0%) and 1,658 out of 1,709 (97.2%) protein-coding genes were identified in strain SICAUCC 16–0001 and SICAUCC 23–0140, respectively, using the BUSCO data from the *Ascomycota* lineage, indicating good quality gene annotation (Supplementary Table S3). The GC content was determined to be 40.3 and 40.2% across the genome and 52.96 and 52.97% in coding sequences in *N. sichuanensis* strain SICAUCC 16–0001 and SICAUCC 23–0140, respectively (Table 1). The predicted gene sets were then annotated using multiple databases. A total of 9,723 (94.50%, *N. sichuanensis* SICAUCC 16–0001) and 9,674 (93.80%, *N. sichuanensis* SICAUCC 23–0140) genes were matched to at least one database. Among them, 9,715 (*N. sichuanensis* SICAUCC 16–0001) and 9,666 (*N. sichuanensis* SICAUCC 23–0140) genes were annotated to the NR database, accounting for 94.42 and 93.73% of the total number of genes, respectively.

The genome sizes of the two *Neostagonosporella sichuanensis* isolates were similar to the published genome of *Setophoma terrestris* in the same family (49.0 Mb), and larger than that of the other analyzed phaeosphaeriacous species (34.1 to 41.7 Mb). However, the genome density of *N. sichuanensis* isolates is the lowest of all phaeosphaeriacous species analyzed (Supplementary Table S6). Moreover, the repeat contents of *N. sichuanensis* SICAUCC 16–0001 and *N. sichuanensis* SICAUCC 23–0140 were 34.99 and 34.46%, respectively, significantly larger than the other analyzed phaeosphaeriacous species (0.96 to 24.96%) (Supplementary Table S7), suggesting that a larger number of repetitive elements contributed to the larger genome size and the fewer protein-coding genes in *N. sichuanensis*. Additionally, 6,151 and 6,308 simple sequence repeats were annotated in strain SICAUCC 16–0001 and SICAUCC 23–0140, respectively. These data will provide valuable genetic markers to assist future breeding programs for *N. sichuanensis* (Supplementary Tables S8, S9).

Two *Neostagonosporella sichuanensis* isolates were predicted to be hemibiotrophic and saprotrophic lifestyles using CATAStrophy software (Supplementary Table S10). The other four pathogens, including *Ophiobolus disseminans*, *Stagonospora* sp., *Setomelanomma holmii* and *Setophoma terrestris*, were predicted to be necrotrophic and hemibiotrophic lifestyles.

### 3.2 Expansion of long terminal repeated transposons and efficient repeat-induced point mutations in *Neostagonosporella sichuanensis* genomes

Transposable elements (TEs) comprised 19.08 and 20.09% of *Neostagonosporella sichuanensis* SICAUCC 16–0001 and *N. sichuanensis* SICAUCC 23–0140 genomes, respectively, and were larger than all compared phaeosphaeriacous genomes (0.14 to 18.22%) (Supplementary Table S11). The average number of TEs in the two *N. sichuanensis* strains was 4,496, which is higher than in other phaeosphaeriacous fungi, except *Setophoma terrestris*, which has 4,561. Additionally, long terminal repeated (LTR) elements were significantly more abundant in *N. sichuanensis* (average = 4,018) compared to all other analyzed phaeosphaeriacous fungi.

The RIP mutation pathway is an ancient fungal genome defense strategy that reduces the harmful effects of TEs and other repetitive genomic regions by undergoing transitions from C (cytosine) to T (thymine) bases (Testa et al., 2016). RIP indices were calculated using the TpA/ApT and (CpA + TpG)/(ApC + GpT) indices. A lower (CpA + TpG)/(ApC + GpT) value suggests a stronger RIP mutation, which is in contrast to the TpA/ApT index (Hane and Oliver, 2008). The high value of TpA/ApT (RIP index, 1.55) and the low ratio of (CpA + TpG)/(ApC + GpT) (RIP index, 0.09) in the genomes of the two *Neostagonosporella sichuanensis* strains (Supplementary Table S12) suggested abundant RIP mutations in them. Similarly, we also calculated the two RIP indices of the other nine analyzed phaeosphaeriacous species and found that strong RIP activity possibly occurs in all. Of all the phaeosphaeriacous species analyzed, the *Ampelomyces quisqualis* genome was the most affected by the RIP mechanism, accounting for 25.83% of the genome, followed by *N. sichuanensis*, accounting for 6.98% (*N. sichuanensis* SICAUCC 16–0001) and 6.38% (*N. sichuanensis* SICAUCC 23–0140).

### 3.3 Features of the GC-equilibrated regions and the high-content AT-rich regions in *Neostagonosporella sichuanensis* genomes

**Figure 1:**
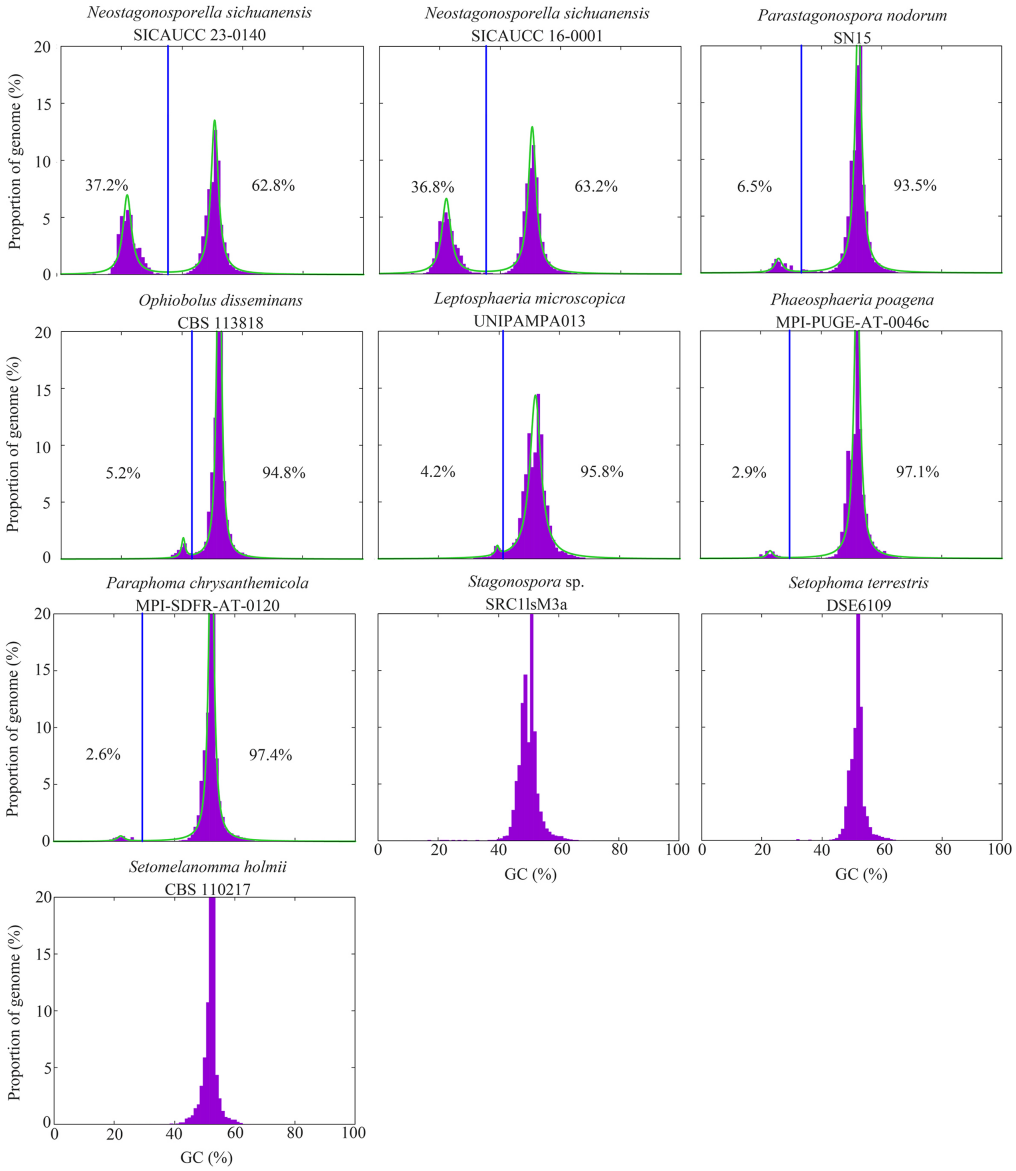
GC content distribution of genomes of the two *Neostagonosporella sichuanensis* isolates, eight other phaeosphaeriacous fungi. Vertical blue lines indicate the GC cutoff points for the classification of genome segments into distinct AT-rich (left) and GC-equilibrated regions (right). The proportion of the AT-rich region in each genome is shown on the left of the vertical blue line, while the proportion of the GC-balanced region of each genome is shown on the right of this line. The green lines represent a mixture of 2 Cauchy distributions fit to the data.

The genomes of *Neostagonosporella sichuanensis* isolates displayed a bimodal GC expression because of the existence of short or long segments of AT-rich regions interspersed within the GC-balanced regions (Figure 1). The proportion of the distinct AT-rich regions of the two *N. sichuanensis* strains (36.8% in *N. sichuanensis* SICAUCC 16–0001 and 37.2% in *N. sichuanensis* SICAUCC 23–0140) was the highest in all analyzed phaeosphaeriacous species, indicating that the two genomes had been extensively invaded by AT-rich regions. Five other species, including *Leptosphaeria microscopica*, *Ophiobolus disseminans*, *Paraphoma chrysanthemicola*, *Parastagonospora nodorum*, and *Phaeosphaeria poagena*, also had two distinct peaks in GC content, but the proportion of AT-rich regions was very low (≤ 6.5%). Additionally, the genome of *Ampelomyces quisqualis* HMLAC05119 was reported to have a 26.6% AT-rich region (Huth et al., 2021). All observed AT-rich regions were gene-sparse compared to regions that were GC-equilibrated (Supplementary Table S13).

**Figure 2:**
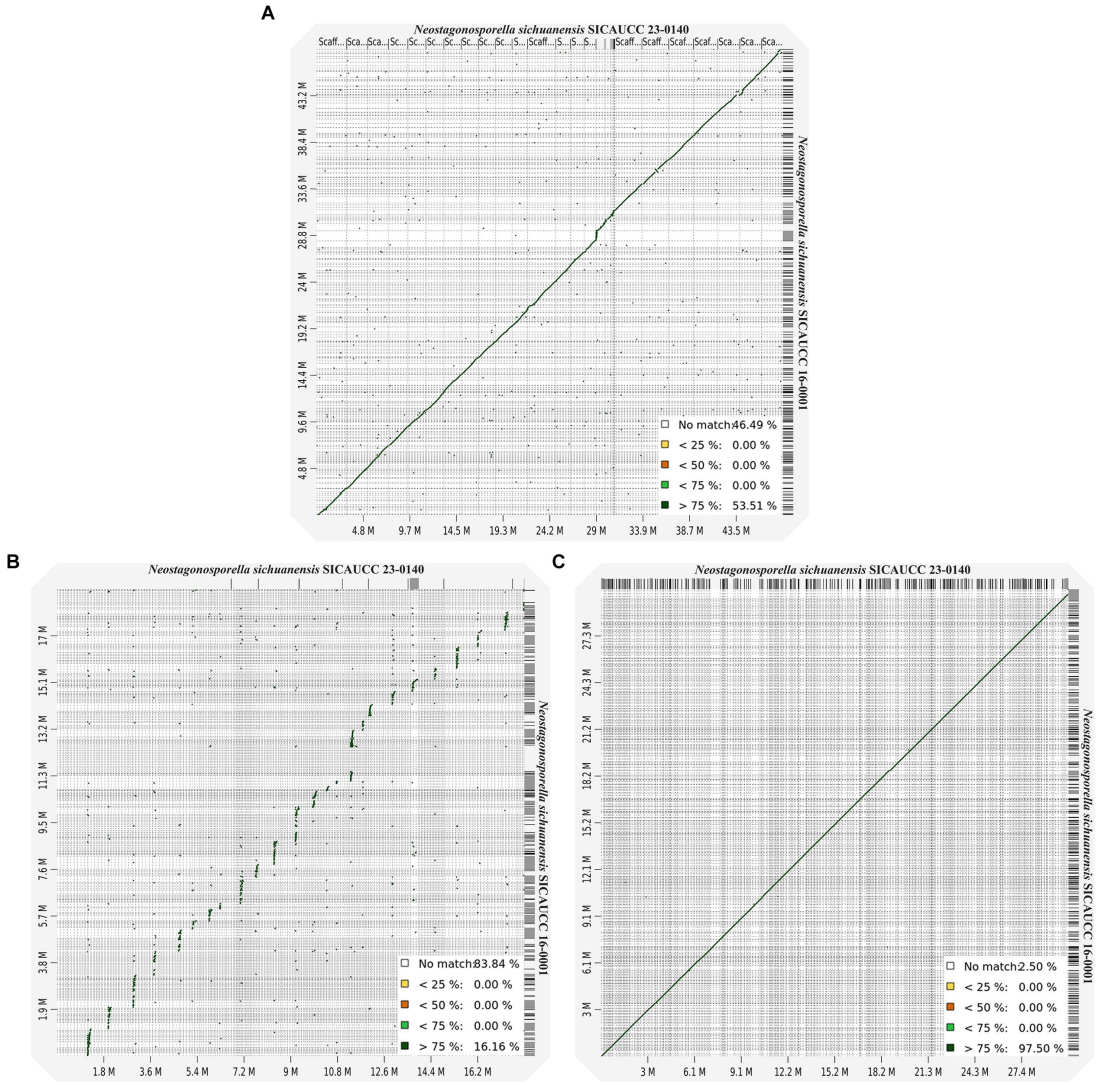
Genomic alignments and synteny between *Neostagonosporella sichuanensis* SICAUCC 16–0001 and SICAUCC 23–0140. **(A)** Whole-genome dot plots. **(B)** AT-rich genome region dot plot. **(C)** GC-equilibrated genome region dot plot.

Next, we aligned whole-genome sequences to further characterize the AT-rich and GC-balanced regions of the genomes of the two *Neostagonosporella sichuanensis* strains. The alignment analysis data (Figure 2A) only revealed 53.51% syntenic blocks that showed >75% similarity between these two strains. The proportion of syntenic blocks was also low in the aligned AT regions (Figure 2B; 16.16% syntenic blocks with >75% similarity), which was the opposite of what we observed in the aligned GC-equilibrated regions (Figure 2C). Taken together, this suggested that the GC-equilibrated regions of the two strains were highly conserved, while AT-rich regions were highly variable. Importantly, this was not observed in other phaeosphaeriacous genomes due to the lack of two or more available genomes from the same species.

### 3.4 Phylogenomic analyses and evolution of *Neostagonosporella sichuanensis* genomes

**Figure 3:**
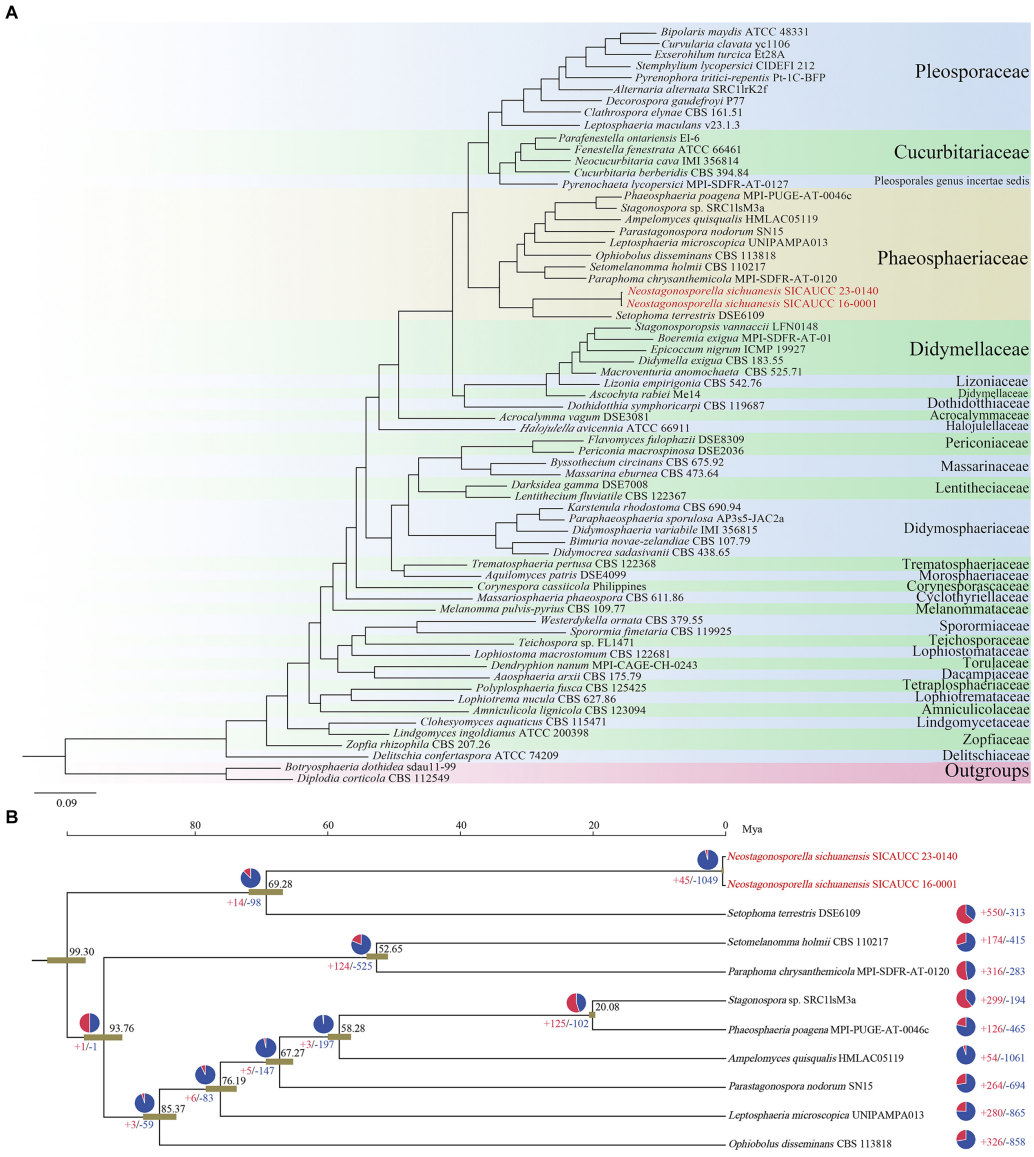
Phylogenomic relationship and evolution analysis of *Neostagonosporella sichuanensis*. **(A)** The phylogenomic relationship of the phaeosphaeriacous species with the other *Pleosporales* fungi analyzed. **(B)** Evolution of the phaeosphaeriacous species analyzed. The black numbers represent divergence time of each node (Mya, million years ago). The pie diagram on each branch of the tree denotes the proportion of gene families undergoing gain (red) or loss (blue) events. The red and blue numbers represent the total number of expansion and contraction gene families, respectively.

The genomes of 62 genera from 28 families and one species with an uncertain genus in *Pleosporales* have been published up to March 2024, according to NCBI and JGI databases. To confirm family-level classifications of the two *Neostagonosporella sichuanensis* isolates and nine other published phaeosphaeriacous species, we constructed a phylogenetic tree by randomly selecting one strain from each genus, together with the unidentified-genus species, two target strains of *N. sichuanensis*, and two outgroup strains (*Botryosphaeria dothidea* sdau 11–99 and *Diplodia corticola* CBS 112549). A total of 26,038 orthogroups were produced in these 66 analyzed genomes with 941,485 proteins using OrthoFinder. Moreover, 1,497 SCO genes across all compared genomes were identified and analyzed to generate a maximum likelihood phylogenomic tree (Figure 3A). All other clades formed with 100% ML support, except the clade (96% support) clustered by *Teichospora* sp., *Westerdykella ornate,* and *Sporormia fimetaria*. The generated phylogenomic tree placed all analyzed species in *Phaeosphaeriaceae* in one clade, confirming the family-level classification results exhibited from the phylogenetic analysis. The species with an unclear genus, *Pyrenochaeta lycopersici* was more closely related to the *Cucurbitariaceae* family at the genome level. *Lizonia empirigonia*, belonging to the *Lizoniaceae* family, was clustered together with didymellaceous species. The results also showed that the two *N. sichuanensis* isolates were more closely related to the pathogenic fungus *Setophoma terrestris*. However, *N. sichuanensis* and *S. terrestris* diverged about 69.28 Mya (Figure 3B), which calibrated with the origin of the *Ascomycota* clade around 500–650 Mya, suggesting a relatively distant speciation event. These two fungi shared a very low genome synteny percentage (Supplementary Figure S2). The analysis of gene family contraction and expansion revealed that the genome of *N. sichuanensis* had 1,049 gene families that underwent contraction, and 45 gene families that underwent expansion. The GO enrichment results showed that the genes of expanded families were mainly enriched in lipid catabolic process, mitochondrion, protein serine/threonine kinase activity and so on. The genes of contracted families were mainly enriched in GTP binding, transmembrane transport, oxidoreductase activity, terpenoid biosynthetic process and so on (Supplementary Table S14). Additionally, we found a notable contraction of several gene families involved in pathogenesis, including those involved in mycotoxin metabolism (aflatoxin biosynthetic process, GO: 0045122), secondary metabolism (indole alkaloid biosynthetic process, GO:0035835; terpenoid biosynthetic process, GO:0016114), sterol biosynthesis and transport (ergosterol biosynthetic process, GO:0006696), and cell wall degradation (xylan catabolic process, GO:0045493; cellulose catabolic process, GO:0030245; response to oxidative stress, GO: 0006979; pectin catabolic process, GO:0045490), and yet no significant expansion of pathogenic gene family was observed.

### 3.5 Analysis of secondary metabolite enzymes of *Neostagonosporella sichuanensis*

**Figure 4:**
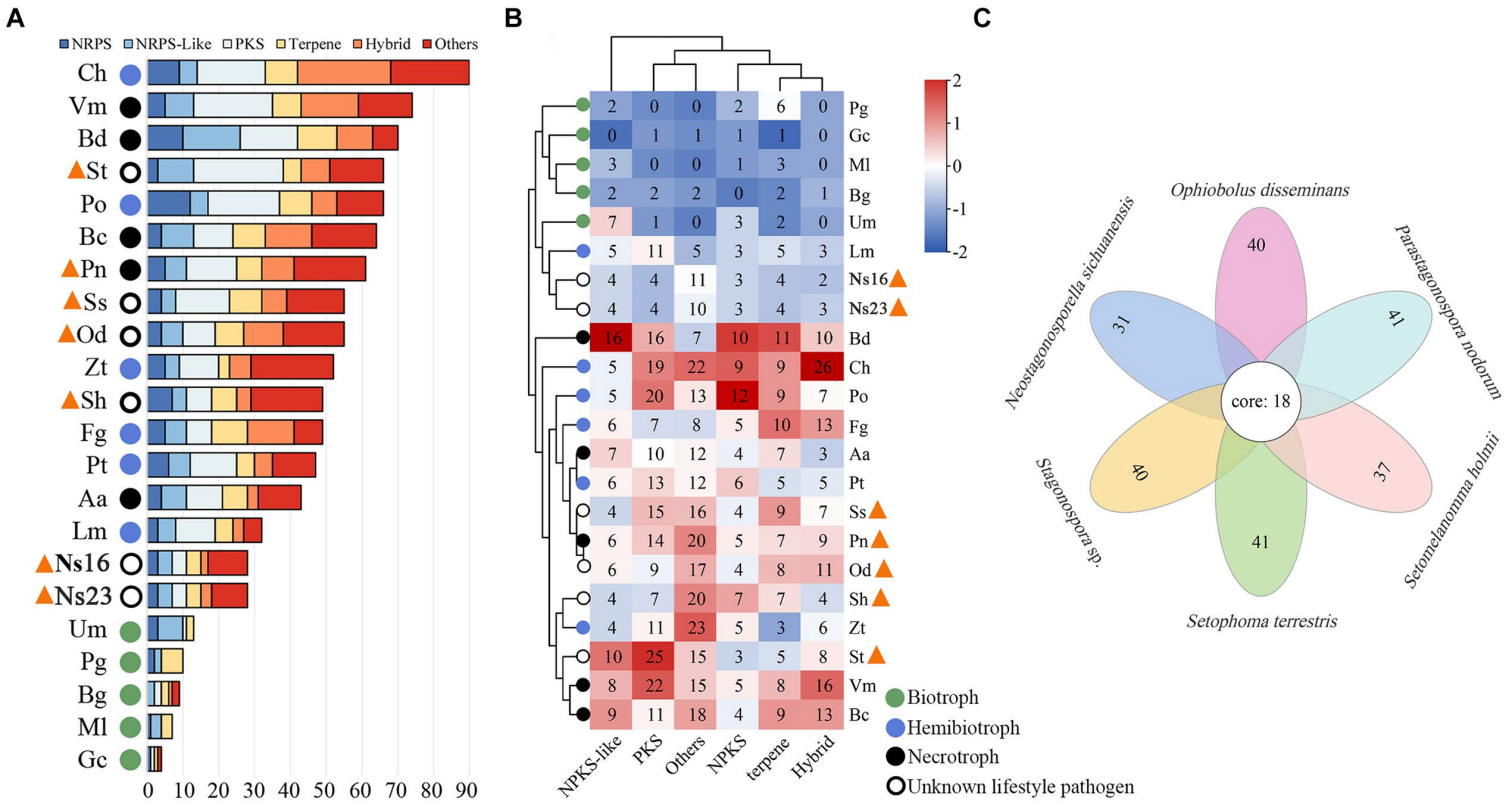
Secondary metabolite gene cluster statistical **(A)** and hierarchical clustering analyses **(B)** of *Neostagonosporella sichuanensis* along with the surveyed phaeosphaeriacous pathogens and other fungal pathogens with different lifestyles. Phaeosphaeriacous pathogens are marked by orange triangles. The numbers in the heatmap cells represented the number of each class of gene clusters. Aa, *Alternaria alternata*; Bg, *Blumeria graminis* f. sp. *triticale*; Bc, *Botrytis cinerea*; Bd, *Botryosphaeria dothidea*; Ch, *Colletotrichum higginsianum*; Fg, *Fusarium graminearum*; Gc, *Golovinomyces cichoracearum*; Lm, *Leptosphaeria maculans*; Ml, *Melampsora larici-populina*; Ns16, *N. sichuanensis* SICAUCC 16–0001; Ns23, *N. sichuanensis* SICAUCC 23–0140; Od, *Ophiobolus disseminans*; Pn, *Parastagonospora nodorum*; Pg, *Puccinia graminis* f. sp. *tritici*; Pt, *Pyrenophora tritici-repentis*; Po, *Pyricularia oryzae*; Sh, *S. holmii*; St, *S. terrestris*; Ss, *Stagonospora* sp.; Um, *Ustilago maydis*; Vm, *Valsa mali*; Zt, *Z. tritici.* Over-represented (+2 to 0) and under-represented (0 to −2) numbers are described as Z-scores for each line in the heatmap. Hybrid represents a gene cluster with no less than 2 core genes, such as PKS-NRPS and PKS-NRPS-like. **(C)** Flower plots analysis of core secondary metabolite genes from genomes of six phaeosphaeriacous pathogens. The secondary metabolite genes of the two *N. sichuanensis* isolates were combined for this analysis.

To analyze the characteristics of secondary metabolite genes, CAZyme genes, secreted proteins, and effectors in *Neostagonosporella sichuanensis*, five phaeosphaeriacous pathogens as well as the other 15 representative fungi including five biotrophic pathogens (*Blumeria graminis* f. sp. *triticale*, *Golovinomyces cichoracearum, Melampsora larici-populina*, *Puccinia graminis* f. sp. *Tritici* and *Ustilago maydis*), five hemibiotrophic model fungus (*Zymoseptoria tritici*, *Pyricularia oryzae*, *Fusarium graminearum*, *Leptosphaeria maculans,* and *Colletotrichum higginsianum*), and five necrotrophs (*Botryosphaeria dothidea*, *Pyrenophora tritici-repentis*, *Valsa mali*, *Botrytis cinerea* and *Alternaria alternata*) were selected for comparative analyses. All genome data are detailed in Supplementary Table S15. A total of 28 secondary metabolite gene clusters were identified from *N. sichuanensis* SICAUCC 16–0001 and *N. sichuanensis* SICAUCC 23–0140, respectively (Figure 4A). This number was lower than all compared hemibiotrophic and necrotrophic fungi, as well as all of the surveyed phaeosphaeriacous pathogens, but significantly higher than those for biotrophic pathogens. Together, the clustering analyses demonstrated that all of the surveyed phaeosphaeriacous pathogens, including *N. sichuanensis,* were grouped with comparable hemibiotrophic and necrotrophic fungi (Figure 4B). Next, we clustered the core secondary metabolite biosynthetic proteins of *N. sichuanensis* and five other pathogens of *Phaeosphaeriaceae* using Orthovenn3. A total of 419 proteins were grouped into 57 gene clusters, leaving only 7 singletons remaining, suggesting that the core biosynthetic proteins across these pathogens are evolutionarily conserved. Further, 31 secondary metabolite protein clusters were found in *N. sichuanensis* and all of them were also found in other *Phaeosphaeriaceae* pathogens (Figure 4C), suggesting the apparent absence of specific genes involved in secondary metabolites produced by *N. sichuanensis*. A total of 18 key biosynthetic protein clusters were distributed in all compared genomes. GO annotation results showed that these clusters were mainly involved in pathogenesis, metabolic processes, transferase activity, mycotoxin biosynthetic processes, phosphopantetheine binding, antibiotic biosynthetic processes, ergosterol biosynthetic processes, lysine biosynthetic processes via aminoadipic acid, isoprenoid metabolic processes, and isoprenoid biosynthetic processes (Supplementary Table S16). The proteins found in six clusters involved in pathogenesis and mycotoxin biosynthetic processes belonged to the oxidase UstYa family of proteins, which have been implicated in the cyclization of toxic cyclic peptides, including ustiloxins in fungi (Wang et al., 2017).

**Figure 5:**
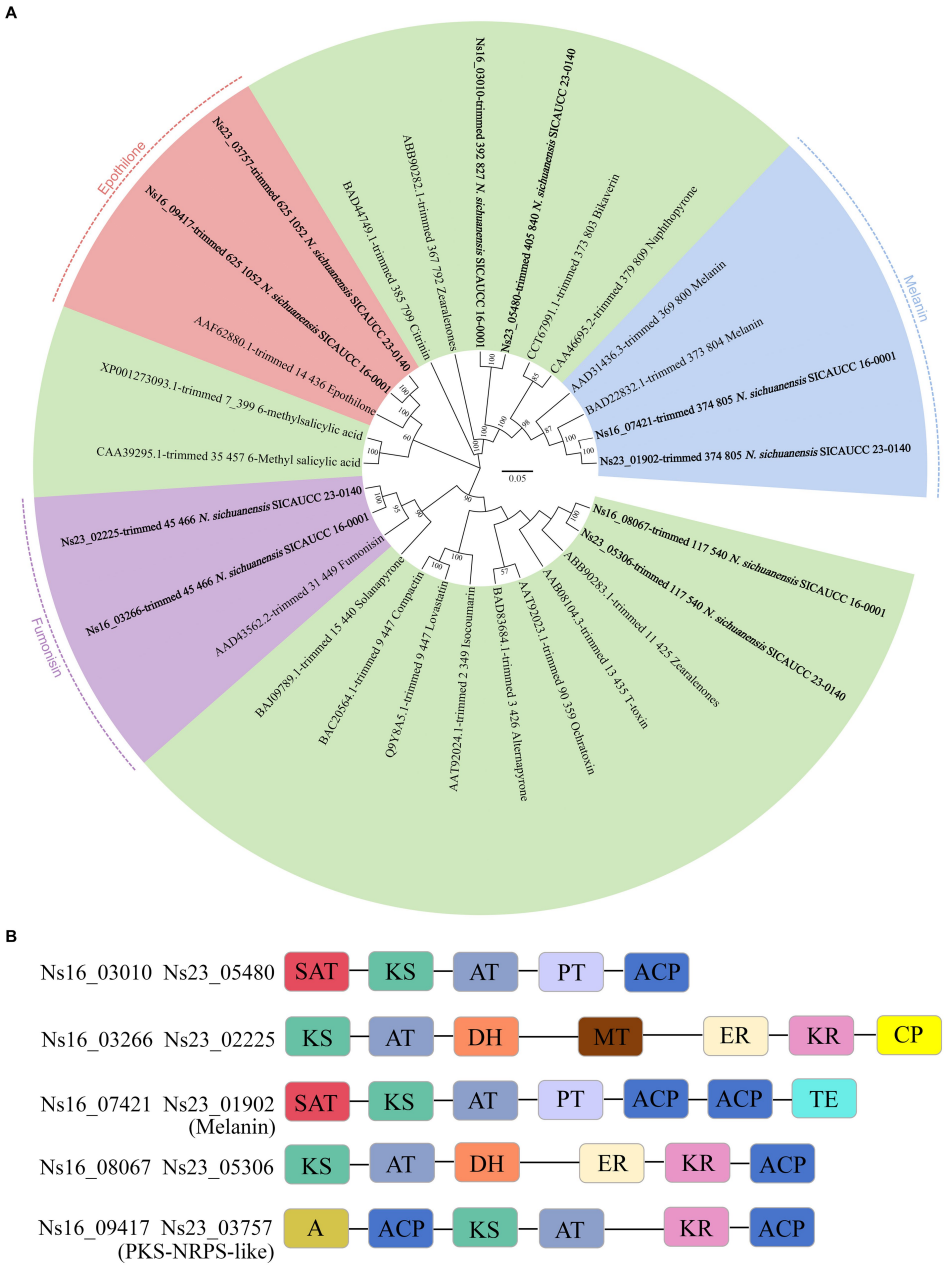
Phylogenetic tree analyses of *Neostagonosporella sichuanensis* KS domains with well-characterized PKS proteins **(A)** and the domains of PKS enzyme **(B)**. The number displayed on each node represents the proportion of 1,000 bootstrap replicates in which they appear. Each domain is displayed in a distinct color. A, adenylation (NRPS-like domain); ACP, acyl carrier protein domain; AT, acyltransferase; CP, phosphopantetheine attachment site; DH, dehydratase; ER enoylreductase; KR, ketoreductase; KS, ketosynthase; MT, methyltransferase; TE, thioesterase; PT, product template; SAT, starter unit acyltransferase.

Fungal PKS is reported to be involved in crucial processes including mycotoxin synthesis for killing host cells in a variety of plants for initial host colonization. To confirm whether any of the PKS genes in *Neostagonosporella sichuanensis* have well-characterized homologs, a maximum likelihood phylogenetic tree was constructed using the predicted PKS amino acid sequences from *N. sichuanensis* and other fungi with well-characterized PKS products (Figure 5A). These analyses revealed that Ns16_07421 and Ns23_01902 were clustered with two PKS enzymes that produce DHN melanin and have 82% sequence similarity with the melanin-producing enzyme (BAD22832,1) from *Bipolaris oryzae* according to BLAST alignment. All of the other PKS proteins from *N. sichuanensis* were shown to have low homology to these compared characterized PKS proteins. However, two of these proteins were clustered with fumonisin-producing PKS protein (Fum1p) and epothilone-producing PKS protein (EpoA-F), with excellent bootstrap values. To further characterize the PKS sequence similarities between *N. sichuanensis* and those of the characterized polyketides, we analyzed the domain structures of *N. sichuanensis* genomes (Figure 5B). The identified proteins that cluster with the PKS enzymes and produce DHN melanin (Ns16_07421 and Ns23_01902) have the following domain organization: SAT-KS-AT-PT-ACP-ACP-TE, which mirrors the domain structures of other PKS enzymes that produce DHN melanin (Noar and Daub, 2016). However, proteins that were clustered together with Fum1p and EpoA-F do not show the same complete domains. While the Fum1p protein contains the KS-AT-DH-MT-ER-KR-ACP domain (Noar and Daub, 2016), both Ns16_03266 and Ns23_02225 only contain the CP domain and lack the ACP domain. EpoA-F consists of two domains; KS-AT-ER-ACP (PKS loading) and Cy-A-Ox-ACP (NRPS module) (Dowling et al., 2016), but Ns16_09417 and Ns23_03757 lack the ER, Cy (cyclization domain), and Ox (a flavin-dependent oxidase domain) domains.

### 3.6 *Neostagonosporella sichuanensis* carbohydrate-active enzymes analysis

**Figure 6:**
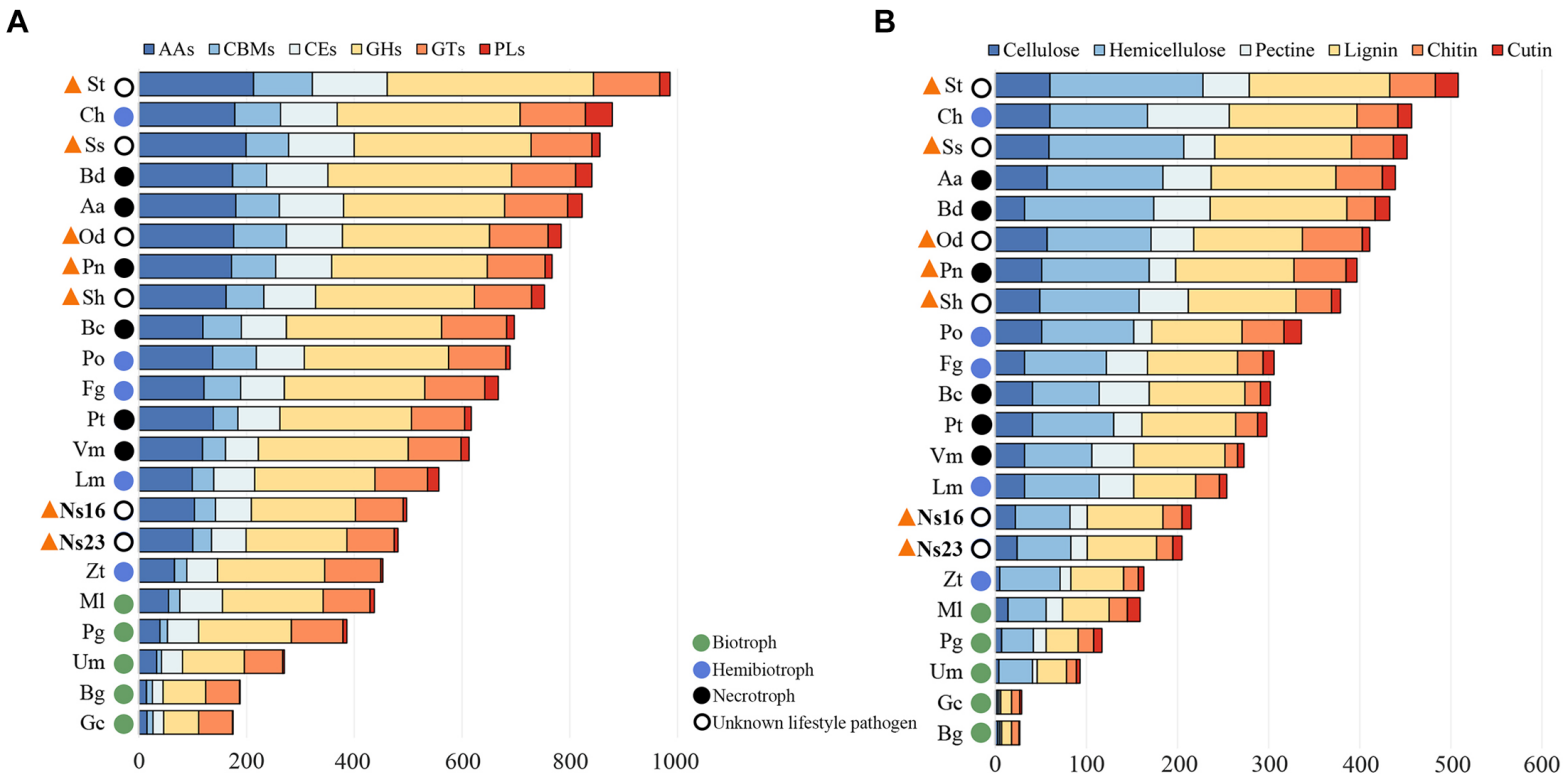
Overall comparison of CAZyme **(A)** and PCWDE expression **(B)** in genomes of *Neostagonosporella sichuanensis* and other fungi with different lifestyles. Selected phaeosphaeriacous pathogens are marked with orange triangles.

In the previous section, we predicted *Neostagonosporella* sichuanesis isolates as hemibiotrophic pathogens based on carbohydrate-active enzymes (CAZymes). Here, we counted the number of CAZymes and plant cell wall degrading enzymes (PCWDEs) modules of *N. sichuanesis* and other organisms. The number of CAZymes and PCWDEs in the two *N. sichuanesis* isolates was lower than that of most hemibiotrophic and necrotrophic pathogens (except for *Zymoseptoria tritici*) and also significantly lower than that of the other five phaeosphaeriacous pathogens (Figures 6A,B). Relatively fewer CAZymes and PCWDEs might be related to expanded considerably transposon elements resulting in fewer encoded genes in genomes of the two *N. sichuanesis* isolates. The same was also true for the genome of *Leptosphaeria maculans*, another hemibiotrophic pathogen extensively invaded by transposon elements. The number of CAZymes and PCWDEs contents in five genome-available pathogens of *Phaeosphaeriaceae* was significantly higher, indicating that they were well equipped with the genes encoding CAZymes and PCWDEs. This was comparable to hemi-biotrophic and necrotrophic pathogens.

**Figure 7:**
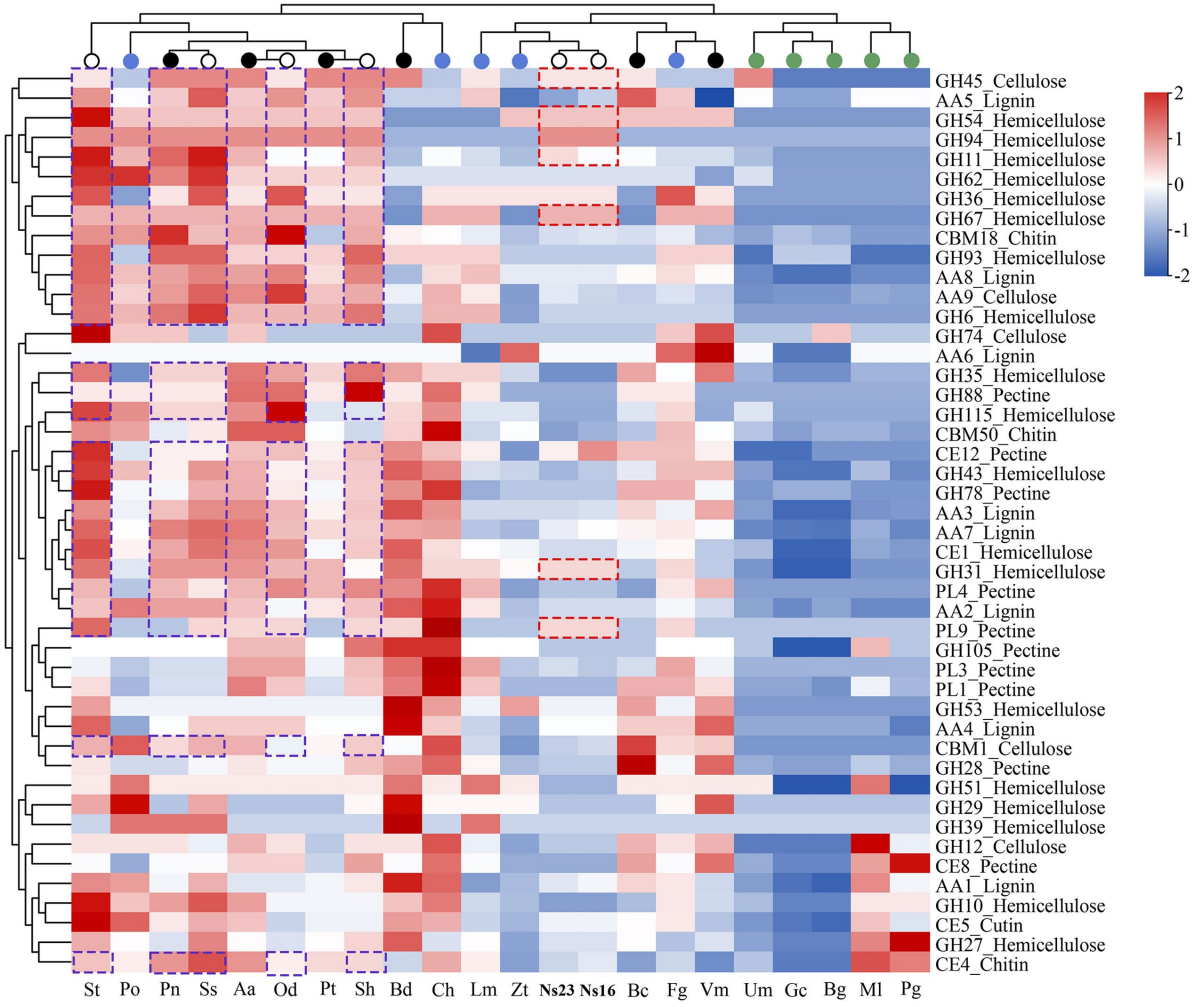
Heatmap clustering of selected PCWDE families from *Neostagonosporella sichuanensis* and surveyed phaeosphaeriacous pathogens, as well as another 15 fungal genomes. Red dashed boxes indicate the expanded CAZyme family in *N. sichuanensis* compared to the other surveyed fungi. Purple dashed boxes highlight the expanded modules in five other surveyed *Phaeosphaeriaceae* pathogens. Over-represented (+2 to 0) and under-represented (0 to −2) numbers are depicted as Z-scores for each family.

Hierarchical clustering analyses of the selected CAZyme families (Yin et al., 2015) showed that the distribution of PCWDEs (cutinases and chitinases included) of *Neostagonosporella sichuanensis* SICAUCC 16–0001 and SICAUCC 23–0140 were most related to two hemibiotrophic pathogens, *Leptosphaeria maculans* and *Zymoseptoria tritici* (Figure 7), a major causal agent responsible for the most destructive septoria tritici blotch disease in wheat. An obvious expansion of three hemicellulose degradation modules in both *N. sichuanensis* strains from the hierarchical clustering, including GH94, GH54, and GH67, suggested a possible relatively strong capacity to degrade hemicellulose. Moreover, two hemicellulose degradation modules (GH31 and GH36), a cellulose degradation module (GH45), and a pectin degradation module (PL9) also displayed a certain degree of expansion in these two strains. This likely explained the importance of these genes and these modules in *N. sichuanensis* pathogenicity and virulence. Additionally, 21 modules (eight hemicellulose, five lignin, three pectin, three cellulose, and two chitin) were also shown to be expanded in at least four phaeosphaeriacous pathogens compared to other analyzed species.

To validate these data, we evaluated the capacity of *Neostagonosporella sichuanensis* SICAUCC 16–0001 and SICAUCC 23–0140 to dissociate cellulose, xylan, and pectin components (Supplementary Figure S3). As expected, both strains grew well with each of the three polysaccharides as the only carbon source. Furthermore, both strains also showed improved growth in the xylan-supplemented medium than in the pectin- and cellulose-containing mediums, which was in line with the increased number of observed hemicellulose degradation modules.

### 3.7 The genetic secretome and effectome of *Neostagonosporella sichuanensis* are most similar to those in necrotrophic and hemibiotrophic pathogens

**Figure 8:**
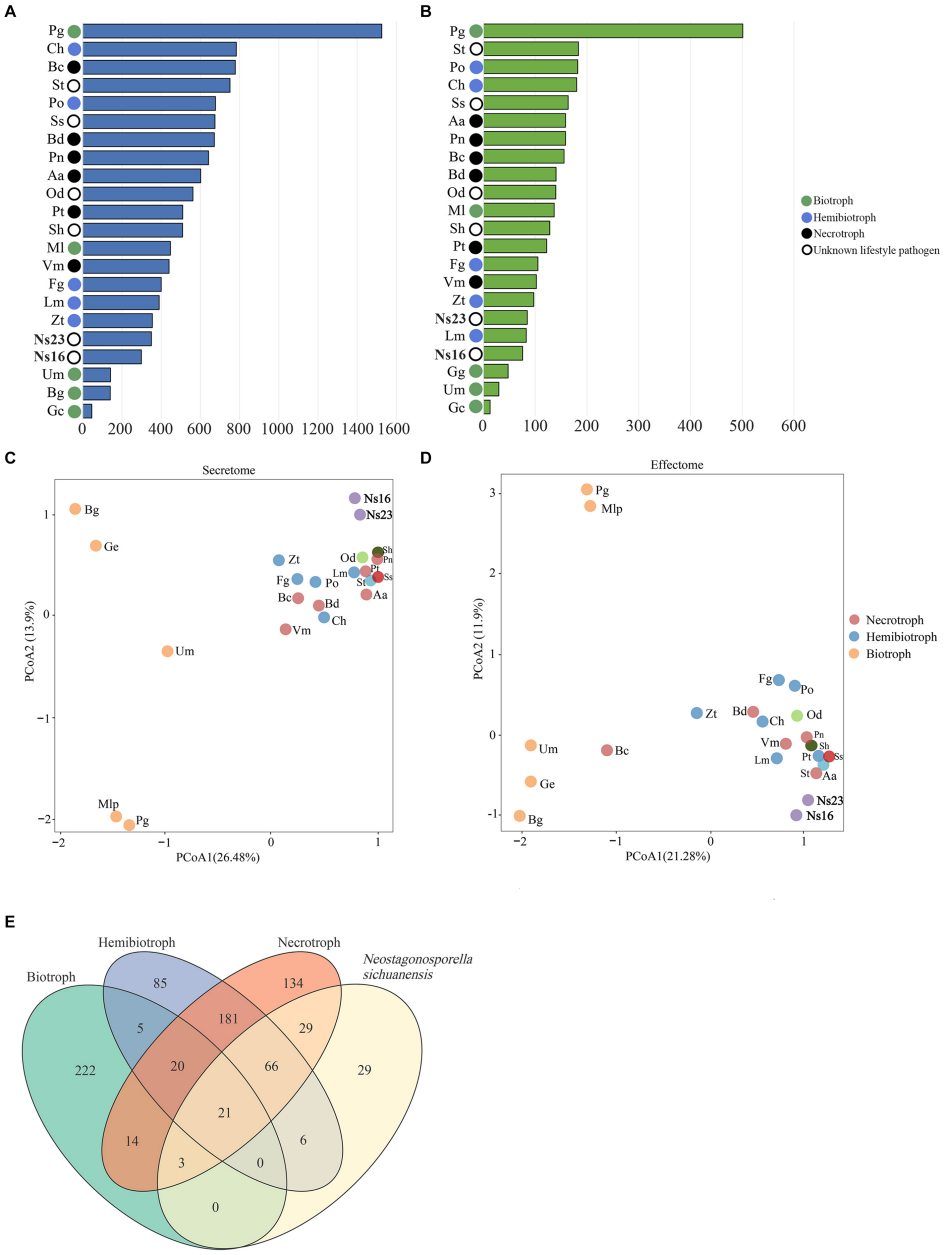
Comparison of secreted proteins **(A)** and effectors **(B)** in *Neostagonosporella sichuanensis*, surveyed phaeosphaeriacous pathogens, and another 15 fungal species with different lifestyles. PCoA plots using the weighted UniFrac distance measure for the secretome **(C)** and effectome **(D)**. Venn diagram **(E)** illustrates core and specific effectome gene families among *N. sichuanensis* and three different lifestyles. The effectors of the two *N. sichuanensis* isolates were combined for these analyses. The number in the Venn diagram represents the specific or core gene families of *N. sichuanensis* and each fungal lifestyle.

The number of predicted secretomes (350 in strain SICAUCC 16–0001 and 299 in SICAUCC 23–0140) and effectomes (169 in strain SICAUCC 16–0001 and 151 in SICAUCC 23–0140) in *Neostagonosporella sichuanensis* were similar to those observed in *Zymoseptoria tritici* and *Leptosphaeria maculans* (Figures 8A,B, respectively). Using OrthoFinder, 1,280 secreted protein orthologous families and 806 effector orthologous families from all species were grouped. The secreted proteins and effectors of genomes of *N. sichuanensis* isolates exhibited an aggregated distribution pattern with that of necrotrophs and hemibiotrophs (ANOSIM: *p* < 0.05) but were distinctively separated from biotrophic pathogens (ANOSIM: *p* > 0.1) according to the result of principal coordinate analysis (PCoA) (Figures 8C,D), thereby indicating more similarities in both to necrotrophic and hemibiotrophic pathogens. The same was true for four other phaeosphaeriacous pathogens with unknown lifestyles, including *Ophiobolus disseminans*, *Stagonospora* sp., *Setomelanomma holmii* and *Setophoma terrestris.*

To investigate the potential mechanism underlying the effector distribution pattern between *Neostagonosporella sichuanensis* and other pathogens with distinct lifestyles, we identified the candidate homologous effector families between *N. sichuanesis* and pathogens with three different lifestyles. The genomes of *N. sichuanesis* isolates have notably more homologous effectors with hemibiotroph and necrotroph fungi analyzed in this work compared to biotroph fungi (Figure 8E). At the same time, we also noticed 21 core effector families were found in the overlapping sets between *N. sichuanensis* and pathogens with distinct lifestyles (Supplementary Table S17). Of these, 12 effector families were annotated as Glyco_hydro_7 (PF00840.21), Glyco_hydro_11 (PF00457.18), Glyco_hydro_28 (PF00295.18), Glyco_hydro_45 (PF02015.17), Glyco_hydro_61 (PF03443.15), Cellulase (PF00150.19), Cutinase (PF01083.23), Ribonuclease (PF00545.21), Peptidase_S8 (PF00082.23), Pectate_lyase_4 (PF00544.20), Egh16-like (PF11327.9), KRE9 (PF05390.12), and a core effector family containing proteins with unknown function (DUF3455; PF11937.9). Eight of the remaining core effector families were lack of observed protein domains. Taken together, we hypothesized that cell wall degrading effectors might be crucial in interactions between *N. sichuanesis* and fishscale bamboos due to their high expression in the core effector families.

## 4 Discussion

Compared to most plant and animal genomes, fungal genomes are usually smaller, with only a few being larger than 100 Mb (e.g., *Cenococcum geophilum* and *Zopfia rhizophila*). The average size of the fungal genomes is about 42.30 Mb, of which the average length of the ascomycetes is 36.91 Mb (Mohanta and Bae, 2015). *Neostagonosporella sichuanensis* genomes are larger than most surveyed phaeosphaeriacous species (except for *Setophoma terrestris* DSE6109, 49 Mb) and the average length of the ascomycetes. The larger genomes of *N. sichuanensis* isolates are speculated associated with extensive invasion of transposable elements (TEs) according to earlier findings for various fungi (Feschotte et al., 2009). Class I transposable elements (retrotransposons) dominate with three families making up 82.8 and 88.1% of TEs in *N. sichuanensis* SICAUCC 16–0001 and SICAUCC 23–0140, respectively. Such a vast-scale invasion by retrotransposons in genomes may be responsible for the low genome GC content and bipartite structure (Rouxel et al., 2011; Dong et al., 2015). The genome of *Ampelomyces quisqualis* HMLAC05119, which has previously been reported to have a bipartite structure (Huth et al., 2021), was also found to have a significant expansion of retrotransposons in this study (Supplementary Table S11). Additionally, the bipartite structures of the two *N. sichuanensis* genomes are very similar to those of *Leptosphaeria maculans*, the first published fungal genome reported to contain a sizable proportion of distinctly AT-rich blocks (Rouxel et al., 2011).

The present study revealed that the degree of collinearity between the AT-rich regions of the two *N. sichuanensis* genomes was markedly low, even though the proportion of AT-rich regions in each genome was comparable. AT-rich regions, the genome regions that have a high proportion of adenine (A) and thymine (T) nucleotides, are often found in overlap with repeat-rich regions, non-coding sequences, regulatory elements, and heterochromatic areas and are less structurally stable than GC-rich regions (Rajewska et al., 2012; Vinogradov and Anatskaya, 2017). AT-rich regions in fungi are often associated with transposable elements or other repeating sequences, resulting in new insertions, excisions, or rearrangements of repetitive sequences (Plissonneau et al., 2018). This dynamic behavior contributes to the rapid evolution of genome architecture, as seen in fungi like *Fusarium oxysporum*, where extensive genome rearrangements are driven by transposable elements, disrupting synteny between strains (Henry et al., 2021). AT-rich regions are also characterized by a high frequency of chromosomal rearrangements, including inversion, translocation and recombination events, disrupting the genetic homogeneity between strains of the same species, leading to genetic differentiation (Dias et al., 2024). Furthermore, the genomes of the two strains under investigation have not been assembled to the chromosomal level, which also explains the observed lack of collinearity in AT-rich regions. RIP mutation is also a crucial process involved in the variable of AT-rich regions in some fungal species. RIP mutations are often limited to the *Ascomycota* subphylum *Pezizomycotina* (van Wyk et al., 2021). In this study, we found evidence of active RIP mutations in the two *N. sichuanesis* genomes (Supplementary Table S18). Additionally, we also provided *in silico* evidence supported RIP activity in other five phaeosphaeriacous species with varying degrees of this bipartite structure, including *A. quisqualis* HMLAC05119, *Leptosphaeria microscopica* UNIPAMPA013, *Ophiobolus disseminans* CBS 113818, *Paraphoma chrysanthemicola* MPI-SDFR-AT-0120, and *Phaeosphaeria poagena* MPI-PUGE-AT-0046c. Nevertheless, the majority of these genomes contain a low number of TEs (Supplementary Table S6), suggesting that run-away genome expansion is typically deleterious. Even though RIP mutations seem necessary for the formation of AT-rich blocks, a genome’s evidence of RIP performance does not always imply that the genome is bimodal (Testa et al., 2016). This is evident in the case of several species in our study, including *Setophoma terrestris* DSE6109, *Stagonospora* sp. SRC1lsM3a, and *Setomelanomma holmii* CBS 110217, of which RIP components were found but not bimodal genomes. Evidence of RIP mutations may potentially originate from past RIP mutations, and this might not be an ongoing process. Repeats are required for the development of AT-rich regions through RIP mutation activity; but, in the absence of further RIPs, the GC content of these sequences may not always differentiate them from the rest of the genome (Testa et al., 2016).

Efficient RIP mutations are thought to prevent the formation of new genes, making the process of large-scale gene family expansion challenging (Meerupati et al., 2013). This may explain why *Neostagonosporella sichuanensis* had far fewer expanded gene families than contracted ones. Gene function simplification and genome compression are important for pathogens to adapt to specific host environments during long-term evolution (O'Connell et al., 2012). The contraction of gene families likely represents a streamlined adaptation strategy that minimizes unnecessary gene retention, enhances genome efficiency and allows pathogens to exploit host resources more effectively while reducing gene redundancy to counteract host defense mechanisms (Raffaele et al., 2010). Genome analysis of the necrotrophic pathogen *Botrytis cinerea* revealed contractions in several gene families involved in secondary metabolite synthesis and plant cell wall degradation, which may indicate that *B. cinerea* optimizes a subset of highly effective pathogenic agents, thereby reducing redundant genes and improving its ability to utilize host resources and respond to host defenses (Dean et al., 2012). The effector gene family associated with pathogenicity in the genome of *Ustilago maydis* showed significant contraction, which likely allows *U. maydis* to streamline its infection process, avoiding the over-activation of host defense responses and thereby enhancing its adaptability (Amselem et al., 2011). It is essential to investigate whether *N. sichuanensis* has evolved a highly specialized infection mechanism tailored to its host fishscale bamboo, thus making a broad gene family unnecessary for diverse environmental conditions or host defenses.

Phylogenomic analysis can enhance the resolution of phylogenetic trees and help support species reclassifications, and further resolve taxonomic difficulties. For example, the genome sequence of *Corynespora olivacea*, a plant pathogen that had previously been incorrectly categorized at the family level, was found to require additional research into its classification (Dal'Sasso et al., 2021). In this study, the two *Neostagonosporella sichuanensis* isolates were placed in the *Phaeosphaeriaceae* clade, confirming the family-level classification results provided by the phylogenomic study. *Lizonia* was placed in *Lizoniaceae* based on morphological study and multi-loci phylogenetic analysis (Boonmee et al., 2017; Wijayawardene et al., 2022), which seems to be controversial. *Lizonia sexangularis* M222 and *Lizonia sexangularis* M179 were placed in *Didymellaceae* based on multi-gene phylogenetic analysis (Mapook et al., 2016). In our study, *Lizonia empirigonia* CBS 542.76 was also clustered in *Didymellaceae* based on phylogenomic analysis. Another pathogen that harms tomatoes and other agronomically important Solanaceous species, *Pyrenochaeta lycopersici*, was unclassified at the family level (Dal et al., 2018; Wijayawardene et al., 2022). Here, we found that *Pyrenochaeta lycopersici* MPI-SDFR-AT-0127 was clustered with four species of the *Cucurbitariaceae* family, revealing that this species was more closely related to the *Cucurbitariaceae* family at the genome level. However, more definitive evidence to clearly define the family of *P. lycopersici* is still lacking. *Leptosphaeria microscopica,* a phaeosphaeriacous endophytic fungus associated with several species of marine macroalgae and mosses in Antarctica (Nordberg et al., 2014), has been controversial since its placement is not supported by morphological and molecular data in currently recognized genera. The current name of *L. microscopica* is recorded as *Phaeosphaeria microscopica* in Index Fungorum website, which is consistent with the result that *P. microscopica* clustered with multiple species of the genus of *Phaeosphaeria* following analyses of sequences of the entire ITS region of 54 species of *Leptosphaeria* and *Phaeosphaeria* (Câmara et al., 2002). However, building a phylogenetic tree with a single gene sequence is limited. In this study, we found that the clade of *P. microscopica* was placed within the *Phaeosphaeriaceae* family, but not clustered with *Phaeosphaeria poagena* in the phylogenomic tree, which may suggest that *P. microscopica* and *P. poagena* are more evolutionarily distant and, therefore, the placement of *P. microscopica* needs further research.

Horizontal clustering analysis of secondary metabolite gene clusters and cell wall degrading enzymes of pathogenic fungi with different lifestyles can help us determine the lifestyle mode of pathogenic fungi with unknown lifestyles (Nagel et al., 2021). In this study, *Neostagonosporella sichuanensis* was inferred to exhibit a hemibiotrophic lifestyle through horizontal cluster analysis of secondary metabolite gene clusters and cell wall degrading enzymes. Approximately 50% of the known fungal phytotoxins are low molecular-weight polyketones that are toxic to plants (Xu et al., 2021). Four PKS genes and one hybrid PKS-NRPS-like gene were predicted in the two *Neostagonosporella sichuanensis* isolates in this study. This number is relatively small compared to predicted PKS genes from other *Dothideomycete* fungi (Noar and Daub, 2016). However, some pathogenic fungi produce polyketides as virulence factors despite having few PKS genes. For instance, *Dothistroma septosporum* can create the polyketide, dothistromin, a key pathogenicity factor, even though only four PKS genes are found in this fungal genome (Noar and Daub, 2016). Even though the genome of *Alternaria brassicicola* contains only six PKS genes, this organism is known to synthesize depudecin, a polyketide that plays a significant role in pathogenicity in host-pathogen interactions (Wight et al., 2009). Thus, the number of PKS genes in fungal genomes cannot be used to measure the significance of polyketides in virulence. However, in this paper we have not investigated the function of these PKS genes in *N. sichuanensis* to prove whether they play a role in the virulence of the pathogen.

We compared the effector diversity of *Neostagonosporella sichuanensis* and pathogens with different lifestyles. As we expected, the effectors of these isolates were clustered with those of necrotrophic and hemibiotrophic pathogens. Moreover, 21 core effector families were examined in the overlapping sets between *N. sichuanensis* and other pathogens with different lifestyles. These data suggest that pathogens with different lifestyles defeat plant immunity with core conserved effectors. We found that putative cell wall degrading effectors occupy a large proportion of the genome of the overlapping sets between *N. sichuanensis* and fungi with different lifestyles, indicating the importance of cell wall degrading effectors for *N. sichuanensis* and these analyzed species. Some cell wall degrading effectors overcome plant cell walls via various glycoside hydrolases, glycosyltransferases, and pectin lyases (Kubicek et al., 2014). For example, the secreted cellulases and endo-β-1,4-xylanases, which belong to GH6 and GH7 families, respectively, are crucial for vertical penetration and horizontal expansion of *Magnaporthe oryzae* (Nguyen et al., 2011). Further, numerous fungal pathogens have evolved the ability to release cutinases, which degrade plant cuticles and may cause the release of damage-associated molecular patterns (Kim et al., 2008; Arya and Cohen, 2022). The function of these core effectors in *N. sichuanensis* is worth further exploration.

## 5 Conclusion

This study provides the first high-quality genome assembly and characterization of *Neostagonosporella sichuanensis*, the causal agent of fishscale bamboo rhombic-spot disease. It also presents the first comparative genomics study encompassing all available genomes of phaeosphaeriaceous species. A significant expansion of transposable elements was observed in *N. sichuanensis* and *Setophoma terrestris*. The genomes of the seven species, including *N. sichuanensis*, *Ampelomyces quisqualis*, *Leptosphaeria microscopica*, *Ophiobolus disseminans*, *Paraphoma chrysanthemicola*, *Parastagonospora nodorum*, and *Phaeosphaeria poagena*, exhibited two distinct peaks in GC content. *In silico* evidence of active RIP mutations was identified across all analyzed phaeosphaeriaceous species. These phaeosphaeriaceous pathogens, including *N. sichuanensis*, *Ophiobolus disseminans*, *Setophoma terrestris*, *Stagonospora* sp., and *Setomelanomma holmii* exhibited hemibiotrophic or necrotrophic genomic characteristics. Most of these pathogens exhibit significantly higher content of secondary metabolism enzymes, carbohydrate-active enzymes, and plant cell wall-degrading enzymes than biotrophic pathogens, except *N. sichuanensis*, which shows only slightly higher content than biotrophic pathogens. Moreover, the secreted proteins and effectors of genomes of the *N. sichuanensis* isolates exhibited an aggregated distribution pattern with that of necrotrophs and hemibiotrophs but were distinctively separated from biotrophic pathogens. These results have improved our understanding of the genomes of *N. sichuanensis* and other analyzed phaeosphaeriaceous species. For future studies, we recommend using *N. sichuanensis* strain SICAUCC 23–0140, which has higher assembly quality, as the reference genome to provide a more reliable foundation for subsequent functional genomics research. Although expanding genome analysis of phaeosphaeriaceous species offers a powerful approach to analyzing whole-genome characterization and predicting extensive pathogenic genes of *N. sichuanensis*, experimental verification of functions of these genes in pathogenic processes is still needed. Additionally, expanding research to the transcriptomic, proteomic, and metabolomic levels is crucial for gaining a comprehensive understanding of the pathogenicity mechanisms of *N. sichuanensis*.

## Data Availability

The datasets presented in this study can be found in online repositories. The names of the repository/repositories and accession number(s) can be found in the article/Supplementary material.

## Footnotes

^1^ https://www.bioinformatics.babraham.ac.uk/projects/fastqc/

^2^ https://fungismash.secondarymetabolites.org/#!/start
