# Supplementary material for: Genome-wide survey of the bipartite structure and pathogenesis-related genes of Neostagonosporella sichuanensis, a causal agent of Fishscale bamboo rhombic-spot disease
Source: Front Microbiol. 2024 Sep 18;15:1456993. doi: 10.3389/fmicb.2024.1456993 (PMC11444983; doi:10.3389/fmicb.2024.1456993)
Supplement: Supplementary file 1 [file Data_Sheet_1.zip › Supplementary Figure.pdf]

Supplementary Material

1 Supplementary Figure 1

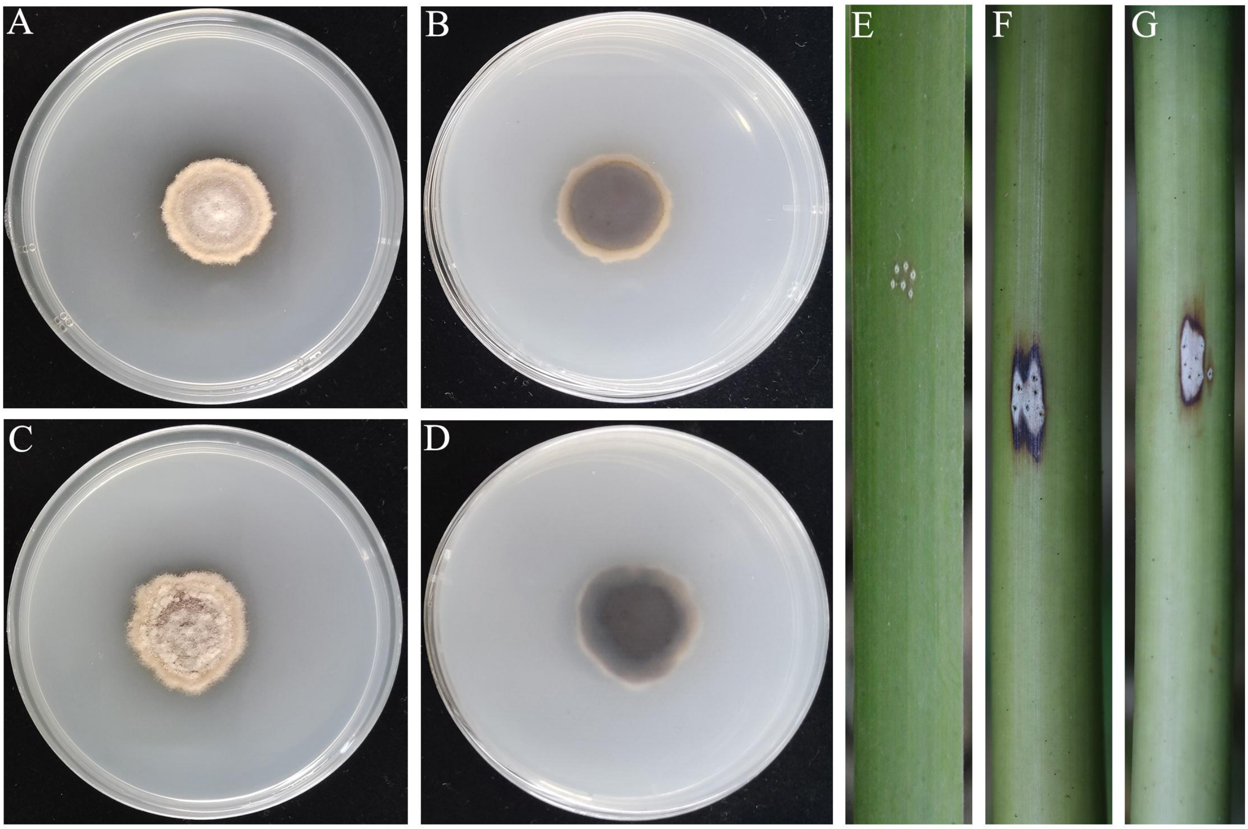

Supplementary Figure 1. Images of *Neostagonosporella sichuanensis* SICAUCC 16-0001 (A-B) and SICAUCC 23-0140 (C-D) colony after growing with single spore on PDA medium for 30 days and the photos of fishscale bamboo after being infected with sterile distilled water as control (E) and conidial suspension of *N. sichuanensis* SICAUCC 16-0001 (F) and SICAUCC 23-0140 (G) for 15 days

Note: Pathogenicity was assessed using needle inoculation. Conidia were harvested and prepared into 1×10^6 spores/mL suspension. One-year-old healthy bamboos were selected, and the surfaces of the bamboo stems were washed three times with sterile water, followed by 75% ethanol for 1 minute and 30 seconds and sterile water three times. Sterile needles were used to create wounds on the surface of the bamboo stems, which were then inoculated with the spore suspension. The inoculated areas were covered with plastic bags to maintain humidity. Wounds were inoculated with sterile water as control. Each treatment was performed in triplicate. Disease incidence was monitored daily following inoculation.

2 Supplementary Figure 2

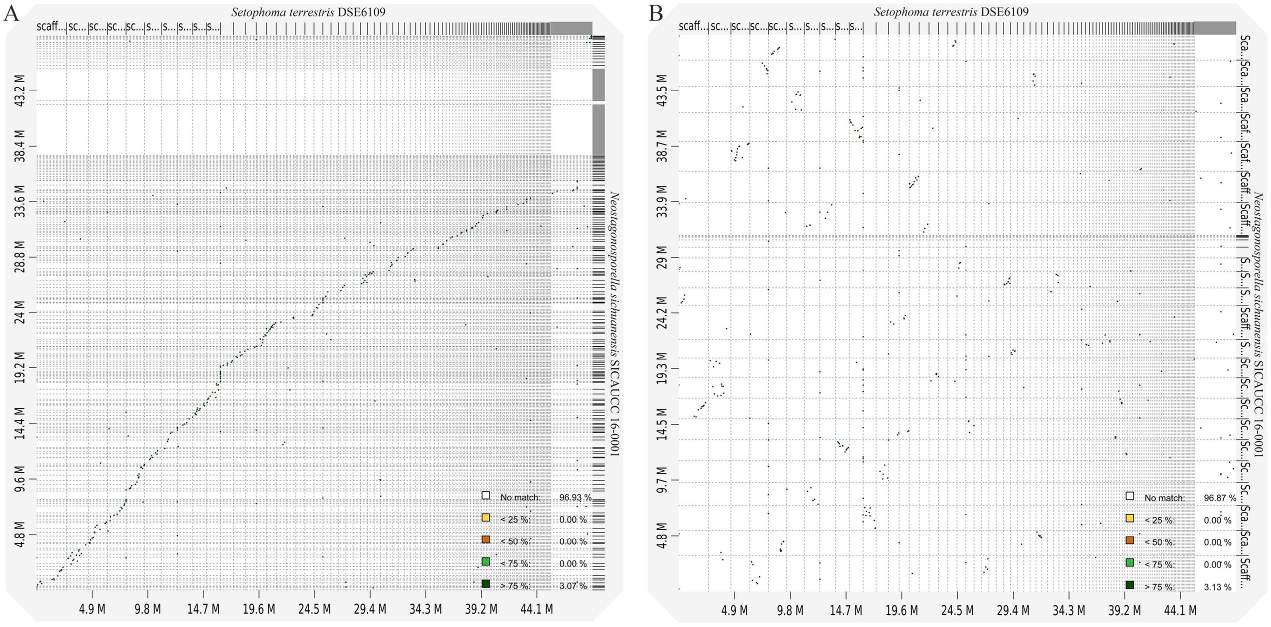

Supplementary Figure 2. Genomic alignments and synteny between *Neostagonosporella sichuanensis* and *Setophoma terrestris*.

3 Supplementary Figure 3

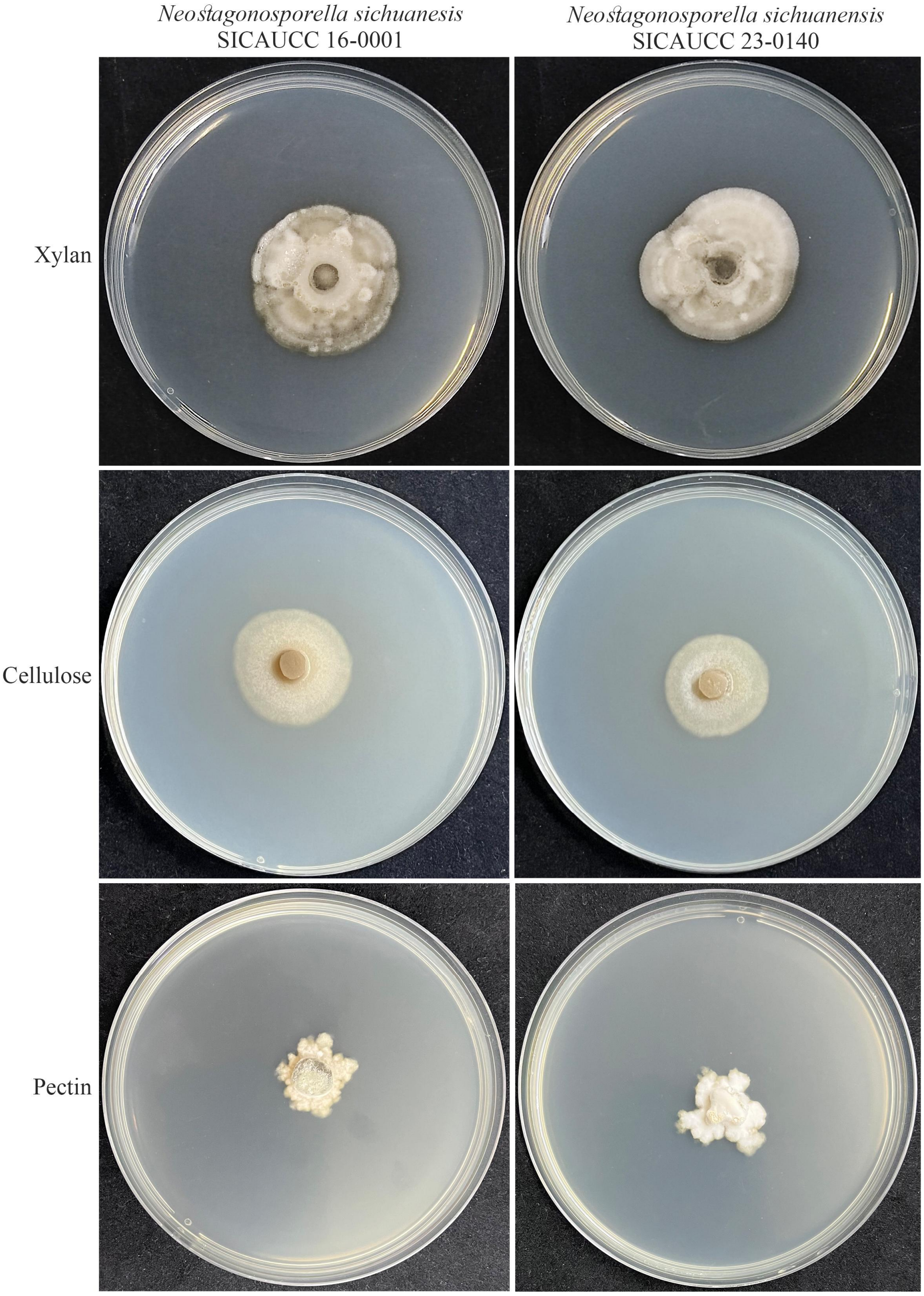

Supplementary Figure 3. Comparison of growth test on different component of cell well of *Neostagonosporella sichuanesis* SICAUCC 16-0001 and *Neostagonosporella sichuanensis* SICAUCC 23-0140

Note: The basic MM medium (1g/L NH_4NO_3, 2g/L KH_2PO_4 0.25 g/L KCl, 0.01g/L $FeSO_4 \cdot 7H_2O$ and 0.1 g/L $ZnSO_4 \cdot 7H_2O$) was supplemented with 10 g/L xylan, microcrystalline cellose (MCC), or pectin. The fungi were incubated for 30 days. The experiments were repeated three times.
